# “Partial Annular Pancreas Causing Obstruction of the First Part of the Duodenum: An Exceedingly Rare Conundrum—A Rare Case Report and Comprehensive Literature Review”

**DOI:** 10.1002/ccr3.70614

**Published:** 2025-07-09

**Authors:** Saurav Jha, Sabin Luitel, Niraj Kushwaha, Sweta Singh, Sulav Kumar Jha

**Affiliations:** ^1^ Department of Radiology Patan Academy of Health Sciences Kathmandu Nepal; ^2^ Chitwan Medical College Chitwan Nepal; ^3^ B.P Koirala Institute of Health Sciences Dharan Nepal; ^4^ Manipal College of Medical Sciences Pokhara Nepal

**Keywords:** annular pancreas, case reports, duodenum

## Abstract

The annular pancreas is one of the very few congenital anomalies associated with gastric outlet obstruction. Usually, it is associated with obstruction of the second part of the duodenum, but to the authors' knowledge, this is the first reported case of the partial annular pancreas obstructing the first part of the duodenum, leading to late presentation of gastric outlet obstruction. As clinicians, we should be vigilant about the causes of abdominal fullness and pain and consider the annular pancreas an important differential diagnosis. It also challenges our predetermined concept of obstruction of the second part of the duodenum secondary to the annular pancreas. Even though the annular pancreas rarely obstructs the first part of the duodenum, it should always be kept in the back of our mind while diagnosing and managing elderly patients presenting with features of gastric outlet obstruction. This paper highlights the atypical presentation of the annular pancreas and how appropriate radiological investigation can clinch the uncommon diagnosis and help in the appropriate management.

## 1 Introduction

Obstruction at the junction of the stomach and duodenum contributes to 5% of all cases of bowel obstruction, highlighting their relatively rare occurrence in comparison with others [1]. The second part of the duodenum is particularly vulnerable to obstruction, whether due to intrinsic factors within the duodenum itself or extrinsic pressures from surrounding structures. One of the rare extrinsic causes of duodenal obstruction is the annular pancreas.

Ecker in 1862 described this infrequent and uncommon anomaly and termed it as Annular Pancreas. The prevalence of annular pancreas has been reported to range from 5 to 15 cases per 100,000 individuals in cadaveric studies, highlighting its rarity. In addition to this, a study using Endoscopic Retrograde Cholangiopancreatography (ERCP) found a slightly higher incidence, estimating it at approximately 1 in 250 individuals [2].

Failure to complete or partial ventral bud rotation during the seventh week of development leads to a complete or partial annular pancreas [3]. In individuals with partial annular pancreas, the annulus, which comprises a band of pancreatic tissue, does not surround the duodenum but extends in the posterolateral, anterolateral, or anterior and posterior to the duodenum, causing its obstruction. Usually, the obstruction secondary to the annular pancreas involves the second part of the duodenum, but the involvement of the first part of the duodenum is extremely rare, and this paper highlights the same rarity.

Most of the patients with annular pancreas are asymptomatic with two peaks of symptomatic presentation, one during infancy and another during the fourth decade of life. During the neonatal period, common clinical manifestations include feeding intolerance, vomiting, and abdominal distension. Adults usually present with features of gastric outlet obstruction such as chronic abdominal pain, nausea, postprandial fullness, and vomiting [4]. An abdominal USG and plain X‐ray usually support the diagnosis of an annular pancreas in neonates. However, in adults, diagnosis is usually made with ERCP and laparotomy. Abdominal CT scans and MRI are also used as a noninvasive method of diagnosis in adults [5].

This case report highlights a rare manifestation of the annular pancreas obstructing the first part of the duodenum with the first presentation at the age of 70 years. Herein, we describe the clinical manifestation of the patient who presented with an age group beyond the usual peak and how we grasped an infrequent congenital anomaly with the help of an appropriate radiological investigation (abdominal CT scan) and isolated a rare site of obstruction associated with it. It provides valuable insight into the dynamics of the annular pancreas, the importance of radiological investigation in diagnosing it, and its variation in an individual patient.

## 2 Case History/Examination/Investigations and Treatment

A 71‐year‐old woman presented with chief complaints of upper abdominal pain and decreased appetite for 2 months. On further inquiry, the patient also complained of vomiting after feed consisting of food particles and was non‐bilious and non‐projectile. She also had a change in bowel habits with alternating loose stools and constipation for 1 month. The patient had similar complaints in the past for which she used to take over‐the‐counter drugs that only used to provide her partial relief with the reemergence of symptoms again after some period of time. The patient denied any history of significant weight loss, prior abdominal surgeries, or chronic use of over‐the‐counter NSAIDs. There was no past history of chronic medical illnesses such as diabetes mellitus, hypertension, thyroid disorders, trauma, and hospital admission for features pertaining to gastric outlet obstruction.

Physical examination was normal except for epigastric tenderness on deep palpation and succussion splash, all of which pointed toward gastric outlet obstruction. With this history and clinical examination findings, we were able to limit our differential diagnosis to conditions such as peptic ulcer disease, gastric cancer, congenital duodenal obstruction, chronic pancreatitis, duodenal/jejunal strictures, small bowel obstruction, and GIST.

All laboratory investigations including complete blood count, serum amylase, serum lipase, serum electrolyte level, and urine RME were normal, which helped in ruling out differential diagnosis such as chronic pancreatitis. Upper GI endoscopy revealed features suggestive of gastric outlet obstruction, and a biopsy was also sent for gastric tissue. There were no signs of ulcer or any mass on UGI endoscopy, ruling out differentials such as gastric cancer. An abdominal CT scan was then planned for her persistent symptoms, which showed no signs of GIST and malignancy.

**FIGURE 1:**
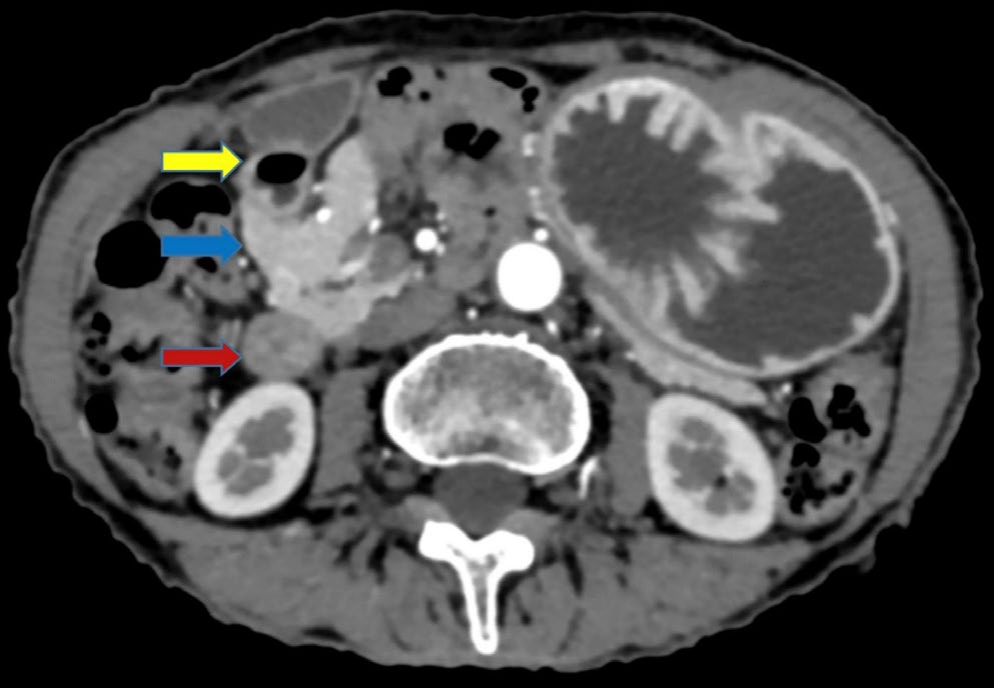
Axial CECT image in arterial phase showing partial encasement of first part of duodenum by head of pancreas indicated by yellow and blue arrow respectively. Normal appearance of second part of duodenum indicated by red arrow.

**FIGURE 2:**
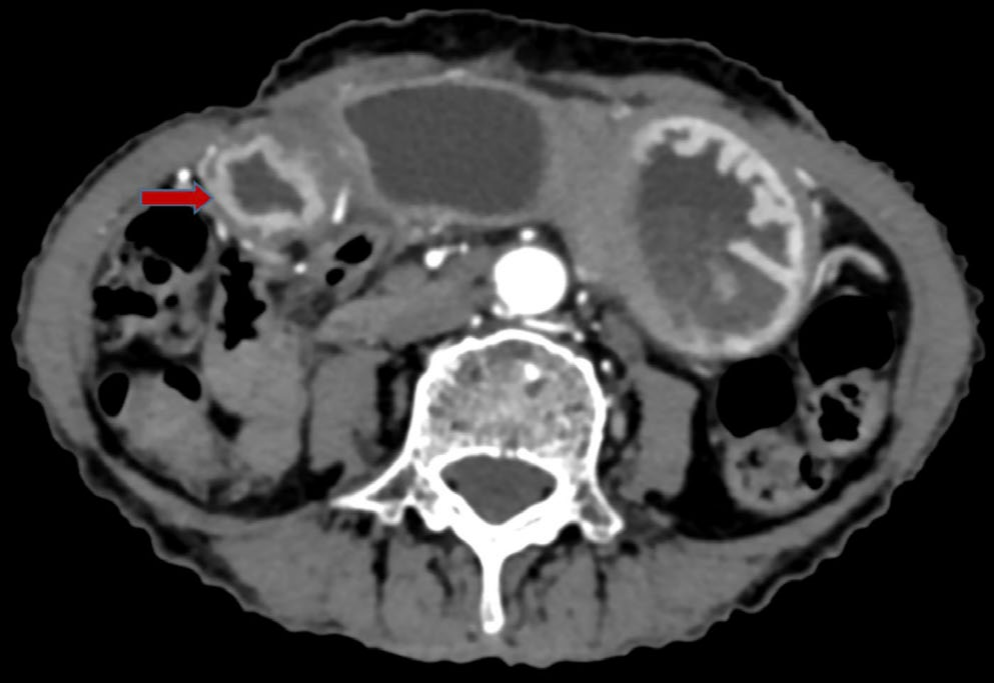
Axial CECT image in arterial phase showing symmetrical gastric wall thickening at the pylorus of stomach with enhancing mucosa indicated by the red arrow.

**FIGURE 3:**
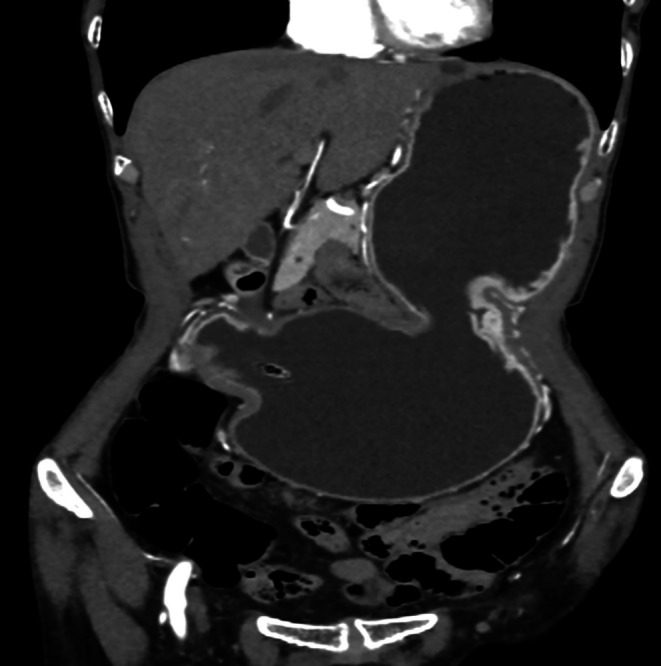
Coronal CECT image in arterial phase showing gross distension of stomach.

**FIGURE 4:**
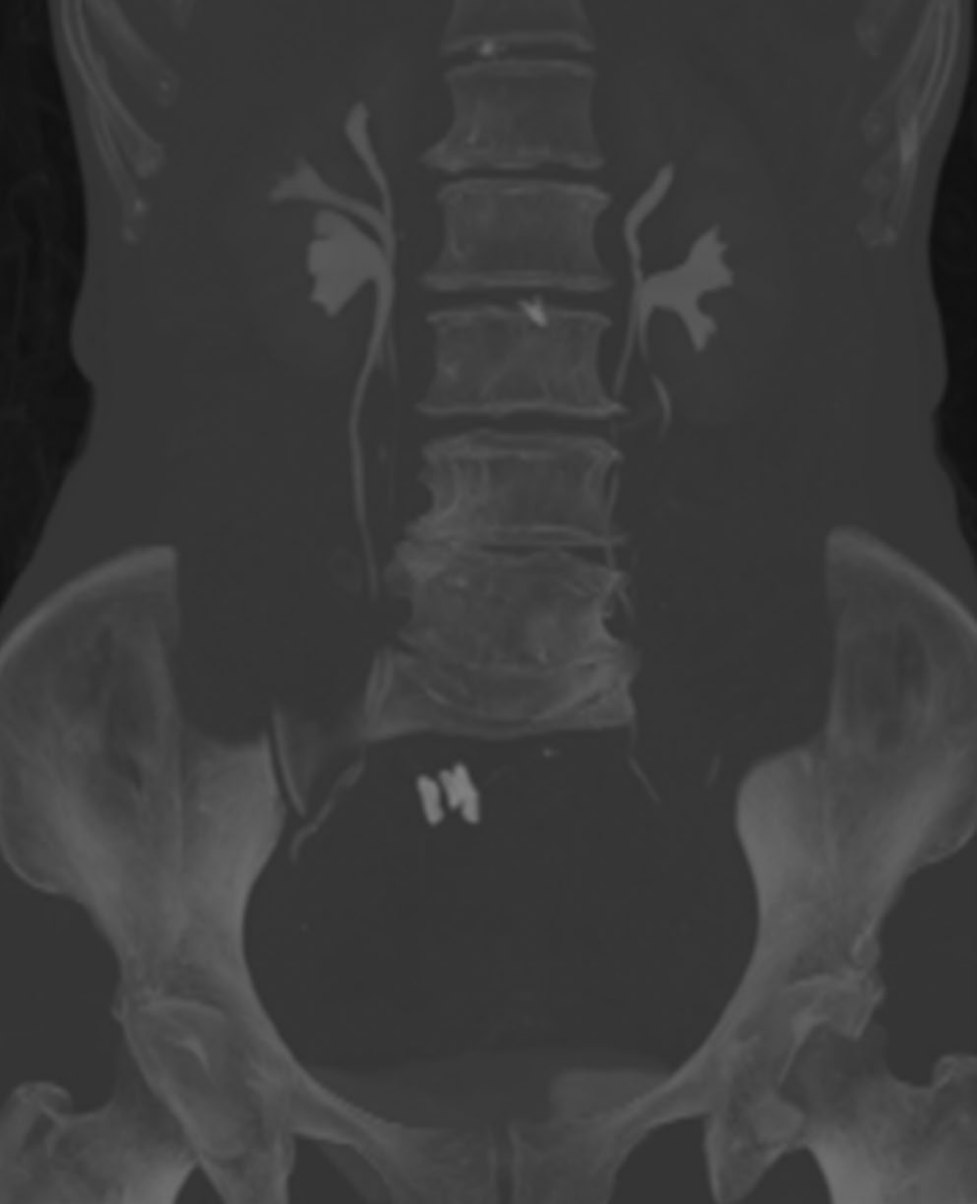
Incidental finding at coronal maximum intensity projection in delayed phase showing duplication of bilateral ureter.

Gastric biopsy revealed *Helicobacter pylori‐*associated chronic active gastritis with intestinal metaplasia with no sign of dysplasia or malignancy. A CT scan of the abdomen revealed a pancreatic head partially encircling the first part of the duodenum, causing its luminal narrowing, while the second part of the duodenum had a normal appearance (Figure 1). The pylorus of the stomach, measuring up to 12 mm, had a diffuse, symmetrically thickened wall, with preserved gut architecture, and a distended stomach was noted (Figures 2 and 3). An incidental finding of duplicated bilateral ureters draining separately into the urinary bladder was also noted (Figure 4). Patients' history, clinical examination, and radiological imaging were discussed with the surgical team, and the patient was then planned for duodenoduodenostomy. The postoperative period was uneventful, and no active complaint was present during the follow‐up period. Coexistent *H. pylori* gastritis was also linked to exacerbation of symptoms and was managed with triple therapy during the course of the hospital stay and follow‐up period.

## 3 Discussion

In adults, the annular pancreas is among some of the rare congenital anomalies associated with upper gastrointestinal obstruction. First described by Tiedemann in 1818, its rarity was supported by Vasconcelos and Sadek's autopsy finding, which revealed only a single case in 22,243 autopsies [6]. Fusion anomalies associated with the pancreas include the annular pancreas, pancreatic divisum, and portal annular pancreas, with the latter being the rarest among all of them.

During the fifth week of development, the foregut gives rise to outpouchings called the dorsal and ventral pancreatic bud, which are the primordial tissues laying the foundation for the development of the pancreas. By the seventh week of development, the ventral bud rotates clockwise and passes behind the duodenum, which then fuses with the dorsal bud. The ventral bud then gives rise to the uncinate process and head of the pancreas, whereas the dorsal bud gives rise to the body and tail of the pancreas [2]. Various theories have been postulated for the development of the annular pancreas, of which the most accepted one is Lecco's and Baldwin's theory. Lecco postulated that the ventral pancreatic bud adheres to the duodenal wall and there is a subsequent failure of its rotation leading to the development of an annular pancreas, whereas Baldwin reported about the persistence of the left pancreatic bud which leads to the complete encirclement of the duodenum by pancreatic tissues and subsequently the development of the annular pancreas [7].

Mostly annular pancreas presents during the neonatal period with features of duodenal obstruction and is usually associated with various congenital anomalies [4]. Some of the common associated anomalies include Down syndrome, situs inversus, mobile right colon, and genitourinary abnormalities such as ambiguous genitalia and dysplastic kidney [4]. Approximately half to two‐thirds of adult patients with annular pancreas are asymptomatic. Symptomatic adults usually present during the 3rd to 6th decade of life with a wide range of clinical manifestations ranging from pancreatitis (acute or chronic), duodenal obstruction, and sometimes peptic ulcer disease. Sometimes it can present as obstructive jaundice and in rare cases as malignancy. Various causes of gastric outlet obstruction have been linked according to the site of obstruction. For instance, volvulus affects the distal stomach, peptic ulcer disease affects the pylorus or the first part of the duodenum, annular pancreas affects the second part of the duodenum, and superior mesenteric artery syndrome usually affects the 3rd part of the duodenum [8]. The D2 segment is by far the most common site of obstruction by the annular pancreas, but the involvement of the first part of the duodenum is a scarce phenomenon, and this paper intensifies this very concept. Along that, it also highlights the radiological features that might be unexpected in an adult patient with features of gastric outlet obstruction.

Features of gastric outlet obstruction in elderly patients arouse suspicion for malignancy and this is one of the closest differential diagnoses in elderly patients for annular pancreas. Other than that, peptic ulcer disease also remains one of the mimickers for annular pancreas in elderly patients. The annular pancreas may be complete or partial depending upon the degree to which pancreatic tissue encircles the duodenum. In the partial annular pancreas, pancreatic tissues partially surround the duodenum giving rise to a crocodile jaw appearance [2].

Neonates with features of feeding intolerance, abdominal distension, and vomiting should be suspected for annular pancreas. Diagnosis in neonates is usually supported by a plain abdominal radiograph which depicts a classical double bubble sign, but this finding is not diagnostic of the annular pancreas as it is also associated with other conditions such as duodenal atresia and intestinal malrotation. Supportive findings suggestive of duodenal obstruction in neonates require no further investigation, as surgical correction is needed for all patients in this age group with complete or partial duodenal obstruction [4].

Diagnosis in adults is made with the help of a spectrum of investigations ranging from noninvasive methods such as abdominal CT scans, MRI, upper gastrointestinal series to invasive methods such as ERCP and laparotomy [9]. An abdominal CT scan in the case of a complete annular pancreas usually shows a complete ring of pancreatic tissue surrounding the duodenum, which may be circular, triangular, or may show the sandwich sign. In patients with partial annular pancreas, pancreatic tissue may extend posterolateral or anterolateral direction to the duodenum or anterior and posterior to the duodenum in a crocodile jaw configuration. The presence of pancreatic tissue posterolateral to the duodenum has high sensitivity and specificity in comparison to the anterolateral direction of pancreatic tissue, which is less specific in identifying partial annular pancreas. MRI has superiority to CT scan in identifying annular ducts or associated anomalies such as pancreatic divisum [2]. The upper gastrointestinal series usually reveals eccentric or concentric narrowing of the obstructed part of the duodenum and symmetric dilation of the proximal duodenum. Reverse peristalsis of the duodenal segment proximal to the annulus and dilation of the duodenum distal to the annulus can be seen in some cases [10].

While the radiological investigation has gained much technical advancement, each radiological modality has its limitations. Abdominal CT scans in infants are limited by the scarcity of fat, which serves as a reference for enhancement. In contrast, in adults, its use is limited by the narrowness of the ring, and the pancreatic ring may lie intramurally in the duodenum without any plain existing in between. In addition to this, the use of ERCP is limited by its risk of causing pancreatitis, and the use of MRCP requires a dilated ductal system [2].

Usually, the radiological imaging finding that has been described in the literature about gastric outlet obstruction secondary to the annular pancreas mostly correlates with the obstruction in the second part of the duodenum. Still, our patient presented with features suggestive of gastric outlet obstruction with luminal narrowing in the first part of the duodenum. With this case report, we want to highlight the atypical presentation of the annular pancreas in the atypical age group with a rare site of obstruction. It aims to provide insight into D1 obstruction caused by a rare congenital anomaly and how radiologists should keep a vigilant vision while analyzing patients with gastric outlet obstruction. We herein objectify the importance of radiological investigations such as abdominal CT scans in guiding the early diagnosis and management of patients with gastric outlet obstruction caused by congenital anomaly in the elderly age group.

## 4 Conclusion

Annular pancreas is one of the few rare congenital anomalies, which should be considered when evaluating patients with nonspecific symptoms of gastric outlet obstruction in the elderly age group. Through a detailed clinical examination and radiological investigation, we could diagnose annular pancreas in a female of 71 years old. Abdominal USG has no role in diagnosis, but the diagnosis seems to be fairly recognized by noninvasive investigations such as CT scans. Herein, we highlight the radiological feature of an uncommon location of obstruction of annular pancreas involving the first part of the duodenum. The imaging finding of crocodile jaw fashion of the pancreatic head or the posterolateral extension of pancreatic tissue to the duodenum are some of the classical features of the annular pancreas. With this case report, we want to contribute to the literature, the radiological imaging findings of the D1 segment obstruction done by the annular pancreas and provide a reference for radiologists facing similar complex scenarios in the future.

## Author Contributions

**Saurav Jha:** conceptualization, writing – original draft. **Sabin Luitel:** conceptualization. **Niraj Kushwaha:** writing – review and editing. **Sweta Singh:** writing – review and editing. **Sulav Kumar Jha:** writing – review and editing.

## Consent

Written informed consent was obtained from the patient to publish this report in accordance with the journal's patient consent policy.

## Conflicts of Interest

The authors declare no conflicts of interest.

## Data Availability

Data will be provided by the corresponding author upon reasonable request. Images have been uploaded in separate files.
